# Factors Contributing to the Risk of HIV Infection in Rural School-Going Adolescents

**DOI:** 10.3390/ijerph111111805

**Published:** 2014-11-14

**Authors:** Adedapo Awotidebe, Julie Phillips, Willy Lens

**Affiliations:** 1 Faculty of Community and Health Sciences, Department of Physiotherapy, University of the Western Cape, Private Bag X17, Bellville 7535, South Africa; E-Mail: awotidebe.adedapo@gmail.com; 2 Faculty of Psychology and Educational Sciences, University of Leuven, Tiensestraat 102, B-3000 Leuven, Belgium; E-Mail: Willy.Lens@ppw.kuleuven.be

**Keywords:** HIV infection, rural adolescents, risky sexual behaviors, risk factors, South Africa

## Abstract

The objective of this study was to determine the factors that increase the risk of HIV infection in rural school-going adolescents and young adults. This was a cross-sectional study of 430 secondary school students (47.4% boys and 52.6% girls) from two rural schools in South Africa. Data were collected with a self-administered questionnaire on demographic information, sources of HIV/AIDS information, HIV knowledge, sexual behaviors, communication and negotiation skills, self-efficacy to refuse sex, peer influence and time perspective. Out of 113 (27.2%) participants who reported being sexually active, about 48% reported having had sex before the age of 15 and 42.2% reported penetrative sex with more than one partner in their lifetime. Only 44.8% of them reported consistent and regular use of condoms for every sexual encounter. Peer influence (OR = 3.01 (95% CI = 1.97–4.60)), gender difference (OR = 6.60 (95% CI = 1.62–26.84)) and lack of HIV information (OR = 1.22 (95% CI = 1.03–1.44)) influenced the sexual risk behaviors of the adolescents. Greater numbers of school-going adolescents in rural areas are sexually active. Peer influence, especially in boys, is a factor that increases the preponderance of risky sexual behaviors in adolescents. Positively, adolescents with high knowledge of HIV infection are more likely to use condoms for every sexual encounter. There is a need to strengthen comprehensive sexual health education and youth-friendly HIV prevention strategies to promote abstinence and safe sexual behaviors, especially among boys.

## 1. Introduction

High proportions of adolescents and emerging adults between the ages of 15–24 years in Sub-Saharan Africa are increasingly sexually active and at higher risk of contracting sexually transmitted diseases [1]. This is because risky sexual behaviors, including sexual intercourse before the age of 15 years, multiple sexual partners and sex without condoms, are now rife among adolescents and disproportionately higher in rural areas [1]. Overall, these risky sexual behaviors are predominantly higher in boys compared to girls of the same age, partly because of the high level of testosterone in boys, which increases early disposition to sexual activities [2]. For example, a Behavioural Health Survey Study (BHSS) among the school-going adolescents in eight selected African countries found that 38.1% of boys, compared to 15.8% of girls, reported sexual activities before the age of 15 years [3]. However, condom use, measured by condom use for the last sexual encounter, was lower in girls (73.1%) compared to boys (87.4%) [4]. The lower proportion of girls who use condoms during sexual activities could be related to the fact that many girls have a sexual relationship with men who are 5–10 years older and might not have the needed skills to negotiate safe sex [5]. In addition, adolescents’ brain structures are less developed and less well equipped to make rational judgments on complex sexual relationships, thereby predisposing them to heightened risk [6].

For these reasons, the United Nations Children Fund (UNICEF) [7] continues to highlight youth’s vulnerability to HIV infection and calls for a better understanding of the factors that increase the risk of HIV with the intention to come up with better interventions to modify these behaviors. A number of studies aimed at providing information on what factors increase sexual risk in adolescents offered no clear-cut findings in order to strengthen various HIV prevention interventions. For instance, Peltzer [3] found that parent-child communication about sex and parental connectedness influenced safe sexual behaviors in adolescents. Conversely, Sneed *et al.* [8] and Dilorio, Dudley, Soet and McCarty [9] found no association between parent communication about sex and changes in the behavior of adolescents. Furthermore, several studies found that young people whose friends are sexually active are more likely to initiate early sexual activities, too [10,11]. Similarly, they are more likely to engage in unsafe sex and have more than one partner if they believe most of their friends are having sex [12]. However, Villarruel, Jemmott, Jemmott and Ronis [13] and Rucibwa, Modeste, Montgomery and Fox [14] found no significant associations between youth’s sexual behavior and the sexual behavior of the friends they keep. Similarly, the evidence regarding the association between HIV knowledge and risky sexual behavior is also mixed. Some studies found a positive relationship between high HIV knowledge, HIV testing and consistent condom use in adolescents [15,16] In contrast, some studies found no associations between HIV knowledge and sexual behaviors [17,18].

A population-based study conducted among school-going adolescents from the Eastern Cape of South African indicated that a theory-based, contextually appropriate HIV-reduction program could modify behavior change in the early period of adolescence [19]. However, there is less information on factors that influence the preponderance of sexual risk behaviors in adolescents. The lack of clear-cut findings on the factors that predict risky sexual behaviors and the paucity of studies among youth in rural areas, particularly in the Western Cape, underscore the need for further research in this area. This observation underlines the need to assess the factors that either increase or decrease the risk of youth in rural areas for HIV infection. This is to strengthen the pre-intervention planning of programs in developing a more effective HIV prevention intervention on the HIV-related risky sexual behaviors among youth in rural areas. The aim of this study was to determine predictors of risky sexual behaviors in school-going adolescents aged 13–18 years in the South African rural schools.

## 2. Methodology

### 2.1. Study Setting and Population

The setting for the study was a town in the Witzenberg Local Municipality, the Cape Winelands District of the Western Cape Province. In 2011, the Cape Winelands district had an estimated 13.2% of the total population of the Western Cape, predominantly employed in the agriculture sector [20]. The majority of the learners come from key neighboring towns. There are four high schools; the majority of the learners are Afrikaans speakers and children of farm workers. In 2011, a study from non-metro districts (Cape Winelands, West Coast, Eden, Central Karoo, Overberg) among Grades 8–10 learners demonstrated that the prevalence of adolescents’ risky behaviors, including lifetime alcohol use (67.9%), cannabis use (21%), sexual activity (30.1%) and sex before age 15 (52.1%), was high [21]. This was the basis for choosing the setting for our study.

### 2.2. Study Design and Sample

This study was a cross-sectional study of 430 learners aged 13–18 years who participated in a sport-based intervention on HIV-related risky sexual behaviors from two high schools. This methodological approach provided insight into the prevalence of risky sexual behaviors among rural youth and highlighted the factors that put them at risk. Four schools were eligible and were invited to participate in the study. Letters introducing the study were delivered to the schools, and we contacted the schools’ principals by telephone to provide them with additional information about the study. Two schools agreed to participate in the study. We extended the recruitment of participants for two weeks, and we again approached the other schools for participation; however, we did not get their active, opt-in approval to participate in our study.

The two schools that agreed to participate had a similar number of learners (35–40) and classrooms (4 classrooms in each grade) from eighth grade to twelfth grade. However, the learners in eleventh and twelfth grade were excluded to avoid the disruption of academic activities. This is in accordance with the Western Cape Education guidelines that stipulate that no learners can be involved in any research during their final year of school (twelfth grade). The follow-up data for the intervention program were to be collected the following year, and therefore, eleventh-grade learners were also excluded. From the 24 classrooms in two schools from eighth grade to tenth grade, we paired each classroom with the life orientation subject period, and we used a coin toss to randomly select two classrooms of each from eighth grade to tenth grade. 

### 2.3. Sample Size

The sample size was estimated from 1200 learners in Grades 8–10 aged 13–18 years from the two high schools. We calculated the sample size using a dichotomous outcome variable (sexually active *versus* not sexually active) to predict factors associated with risky sexual behaviors among learners. We used the approach of Browner, Newman and Hulley [22] to estimate the sample size. We estimated the expected proportion of learners to be sexually active at 30% from the previous study [21], and the confidence level of the interval was set at 95% (two-sided). The desired precision (or width) of the confidence was set at 0.1 (two-sided: 0.05 below and 0.05 above). From an estimated sample of 1200 learners from the two schools, using the procedures in [22], this study design would require 323 learners to correctly predict factors associated with risky sexual behaviors among our participants.

### 2.4. Operational Definitions

Sexually active: having a previous history of penetrative sexual intercourse with opposite partners.

Sex communication with parent: having discussed with either parents sexual-related topics on HIV/AIDS and reproductive health.

Contributing factors to HIV: factors that encourage or discourage adolescents to engage in sexual intercourse (e.g., HIV knowledge, self-efficacy, sex communication, negotiation skills).

HIV risk factors: factors associated with the likelihood that an individual has an increased chance of becoming infected with HIV (e.g., sexual activities before the age of 15 years, unprotected sex, more than one sexual partner, *etc.*).

### 2.5. Research Instrument

The data was collected by means of a self-administered questionnaire, which was available in Afrikaans, a predominant language among the learners. 

#### 2.5.1. Demographic Information

This self-constructed scale measured demographic and socio-economic characteristics of the learners. These include age, grade, socioeconomic status (SES) and population group. Socioeconomic status (SES) was measured with the Family Affluence Scale, on the basis of household items, such as the frequency of vacations and the number of people sharing rooms [23]. The SES was categorized as: low, middle and high SES. This approach was deemed to be more reliable than asking for parent’s income from the children. A composite SES score was computed with a 1–8-point scale and categorized on a three-point ordinal scale: low (0–2 scores), middle (3–5) and high SES (6–8) [23].

#### 2.5.2. Sources of Information on HIV/AIDS and Sex Communication with Parents

This self-constructed scale sought to obtain data on important sources of information on HIV and AIDS. The variables measured included important sources of information on HIV and AIDS, sex education, preferred sources of information and if they have ever attended classes on these topics. Communication with parents about sex was measured with a 5-point Likert scale ranging from “very easy” to “not easy”. The questions on communication with parents about sex are four-item questions and measured with a 5-point Likert scale ranging from “very easy” to “not easy”. Examples of items are: “how easy or difficult is it to talk to your mother or father about things that are important to you?”, “Have you ever discussed sex-related matters with your mother or father?”. 

#### 2.5.3. HIV Knowledge Questionnaire (HIV-KQ-18)

The HIV Knowledge Questionnaire (18 item version) (HIV-KQ-18) is a self-administered questionnaire [24] that measures the knowledge of HIV transmission, prevention and misconceptions about HIV infection. It consists of 18 items dichotomized into “true” and “false” or “don’t know”. The total score for each participant was constructed on a continuous scale by adding the number of correct answers ranging from 0 to 18, with higher scores indicating more knowledge of HIV/AIDS. This scale was found to be reliable, and Cronbach’s alpha coefficients of 0.75–0.89 have been reported [24].

#### 2.5.4. Sexual Behaviors

Sexual behaviors were assessed using a self-administered questionnaire adapted from the subscale “sexual activity” of the Youth Risk Behaviour Surveillance System (YRBS) developed by the Center for Disease Control and Prevention (CDC). The YRBS has been used among adolescents both in developed and developing nations, including South Africa [25]. We chose seven questions about sexual behavior for HIV infection from the 2011 YRBS for a standard high school to measure the age of first sexual intercourse, condom use for the first and last sexual contacts and the number of sexual partners. The learners were invited to report whether they were sexually active and, if so, the age of sexual debut, lifetime sexual partners, consistency of condom use and condom use for the last sexual contact. Examples of the questions were: “Have you ever had sexual intercourse?”, “How old were you when you had sexual intercourse for the first time?”, “During your lifetime, with how many people have you had sexual intercourse?”.

#### 2.5.5. Sexual Communication and Negotiation Skills

Learners responded to a series of questions assessing sexual communication and negotiation skills, adapted from the Assertive Sexual Communication Scale [26]. Learners rated each item using a 5-point Likert scale ranging from strongly agree to strongly disagree across 11 items (original psychometrics indicated internal consistency, α = 0.93). Examples of items are: “I would want to know if my partner has ever had an HIV test” and “Most of the time, we do what my partner wants to do”. Higher scores indicate higher skills for communication and negotiation skills.

#### 2.5.6. Self-Efficacy to Refuse Sex

This scale developed by Basen-Engquist and colleagues [27] was designed to assess sexually active and sexually inexperienced learners’ self-efficacy to refuse sex. The scale consists of 4 items with a 5-point Likert scale ranging from very sure to not very sure. An example item is: “...imagine your partner, he or she wants to have sex, but you don’t feel ready; how sure are you that you could keep from having sex until you feel ready”. Higher scores indicate higher perceived ability to refuse sex. The scale was found to be reliable, and a Cronbach’s alpha coefficient of 0.7 has been reported [27].

#### 2.5.7. Peer Norms about Sexual Intercourse (Peer Influence)

This measure from Basen-Engquist *et al.* [27] with four items assessed the influence of perceived peer sexual activities on one’s own sexual behaviors. Possible responses ranged from 1 for “none” and 5 for “all”. Item scores were then added together across the 4 items and total average scores ranging from 1 to 5, with higher scores indicating a greater possibility of being influenced by friends’ sexual behavior. Examples of items are: “How many of your friends believe people of your age should wait until they are older before having sex?” and “How many of your friends believe it is okay now for people of your age to start having sex?”. The original Cronbach’s alpha coefficient for internal consistency was reported as 0.78 [27].

#### 2.5.8. Time Perspective Scale

Zimbardo Time Perspective Inventory (short form) was used to determine the impact of time perspective as a predictor of risky sexual behavior among the participants [28]. The measure we used consists of three sub-scales (present hedonistic, present fatalistic and future perspective). Future orientation was expected to be a negative predictor and present orientation a positive predictor of risky sexual behavior. Answers are given on a five-point Likert scale across 27 items from “very untrue” to “very true”*.* The original Cronbach alpha coefficients for internal consistency were reported as 0.80, 0.74 and 0.81 for present hedonistic, present fatalistic and future perspective, respectively. Examples of items included: “It does not make sense to worry about the future, since there is nothing to do about it anyway”, “Fate determines much in my life” and “My life path is controlled by forces I cannot influence”.

### 2.6. Procedures

#### 2.6.1. Reliability of the Research Instrument

A pilot study was conducted among a group of 30 learners in a high school with a similar demographic profile as the intended study participants. We checked the reliability of scales by calculating the internal consistency (Cronbach’s alpha coefficient to determine the extent to which the items that make up the scale are all measuring the same underlying attribute). The results of the pilot study (Table 1) indicated that all instruments were reliable in our setting, with Cronbach alpha coefficient values above 0.70 [29].

**Table 1:** Psychometric description of scales.

Variables	Range of Scores	No. of Items	Mean (SD)	Cronbach Alpha
HIV knowledge	0–18	18	8.37 (3.06)	0.73
Self-efficacy	1–5	4	3.42 (1.23)	0.82
Communication	1–5	4	4.28 (0.86)	0.81
Peer influence	1–5	4	2.41 (0.91)	0.70
Negotiation	1–5	7	3.39 (0.83)	0.70
FPT	1–5	5	2.72 (0.78)	
HPT	1–5	10	2.80 (0.61)	0.73
Future Time	1–5	11	3.42 (0.69)	0.75

Notes: FPT, fatalistic present time; HPT, hedonistic present time. We combined the presents (FPT and HPT) to get a single Cronbach score.

#### 2.6.2. School-Level Permission

The second author held meetings with the principals from two participating schools and all teachers from selected classes prior to the baseline data collection. Information was given about the aim and nature of the study, everyone’s role and the time commitment to complete the project. Both principals confirmed their permission to conduct the study in their schools.

#### 2.6.3. Parents’ Informed Consent and Learners’ Informed Assent

Teachers in selected classes distributed the study information booklet containing the study information, parents’/guardians’ signed consent forms and learners’ signed informed assent forms. The teachers emphasized their voluntary participation, ability to withdraw from the study and assured each learner’s dignity, respect, privacy, confidentiality and anonymity. Only learners with signed parent consent forms and learners’ assent forms and in class on the day of data collection were included in the study sample. 

Questionnaires were distributed during a life orientation subject period in each selected classroom, and the first author was present to provide instructions and monitor questionnaire administration. Out of an estimated 480 learners from Grades 8–10 from both high schools that were expected to participate in this study, 430 learners who had a signed parent consent form and a learner assent form formed the final sample for this cross-sectional study design.

#### 2.6.4. Ethics Approval

The Ethics Committee at the University of the Western Cape gave ethical approval (Research Proposal Registration Number: UWC/10/1/15). Permission to conduct the study was obtained from the Western Cape Education Department (WCED).

### 2.7. Data Management and Analysis

Data were analyzed with the Statistical Package for the Social Sciences (SPSS^®^) (version 20.0), and the α-value was set to *p* = 0.05. Variables were checked for the normality of the data using the Kolmogorov–Smirnov statistic, and a value greater than 0.05 indicated the normal distribution of the specific variable. Descriptive statistics were expressed as percentages (%) for binary categorical sexual behavior outcomes (sexual experience: active *vs.* inactive; sexual debut: early *vs.* late; multiple sex partners: one partner *vs.* more than one partner; condom use for last sexual intercourse: yes *vs.* no; consistency of condom use: always *vs.* irregular; HIV test: yes *vs.* no). Means (M) and standard deviations (SDs) were performed for selected predictors of sexual behavior outcomes (HIV knowledge, self-efficacy skills, communication skills, negotiation skills, peer influence and time perspective). The chi-square test was to assess gender differences in adolescent sexual behavior outcomes and the independent *t*-test for gender interaction with selected predictors of sexual behavior outcomes. Communication with parents on sexual matters was added as part of the predictors and dichotomized into “easy communication *vs.* not easy communication”. Furthermore, we performed a separate model for binary logistic analysis for each of the sexual behavior outcomes as dependent variables and selected the predictors of sexual behaviors as independent variables (or covariates). We used a forced entry method to determine which of these selected predictors were strongly and significantly associated with sexual behavior outcomes.

## 3. Results 

### 3.1. Demographic Characteristics of Study Sample

The demographic characteristics of the learners are represented in Table 2. Out of the total of 430 learners aged 13–18 years who participated from the two schools, 226 (53.6%) were girls and 386 (94.4%) were from the “mixed” (people of mixed ethnic origin) population group. A significantly higher proportion of girls (37.1%) compared to boys (26.6%) reported easier communication with parents on sex. Learners reported teachers (60.0%) as the most cited source of information on HIV/AIDS, although most learners would have preferred mothers (53.6%) as the primary source of information on HIV/AIDS (Table 2).

**Table 2:** Demographic characteristics of study sample (N = 430). SES, socioeconomic status.

Characteristics	Total Population N (%)	Boys N (%)	Girls N (%)	*p*-value
**Age**				
<15 years	117 (28.4%)	55(28.8%)	62 (28.1%)	0.87
≥15 years	295 (71.6%)	136 (71.2%)	159 (71.9%)	
**Population group**				
Mixed	386 (94.4%)	184 (92.9%)	202 (95.7%)	0.52
Black	10 (2.4%)	5 (2.5%)	5 (2.4%)	
White	8 (2.0%)	5 (2.5%)	3 (1.4%)	
Indian	3 (0.7%)	2 (1.0%)	1 (0.5%)	
Others	2 (0.5%)	2 (1.0 %)	0	
**Grade**				
10	114 (26.6%)	59 (28.9%)	55 (24.4%)	0.41
9	160 (37.3%)	70 (34.3%)	90 (40.0%)	
8	155 (36.1%)	75 (36.8%)	80 (35.6%)	
**SES**				
Low	159 (38.2%)	79 (39.5%)	80 (37.0%)	0.56
Middle	202 (48.6%)	92 (46.0%)	110 (50.9%)	
High	55 (13.2%)	29 (14.5%)	26 (12.1%)	
**Communication (General)**				
Easy	207 (49.3%)	114 (57.0%)	93 (42.3%)	0.003
Difficult	213 (50.7%)	86 (43.0%)	127 (57.7%)	
**Communication (Sexuality)**				
Easy	135 (32.1%)	53 (26.6%)	82 (37.1%)	0.02
Difficult	285 (67.9%)	146 (73.4%)	139 (62.9%)	
**How often did you discuss sexuality communication?**				
Always	55 (14.4%)	23 (12.3%)	32 (16.4%)	0.25
Rarely	327 (85.6%)	164 (87.7%)	163 (83.6%)	
Sources of information on HIV/AIDS *				
Teachers	261 (60.0%)	116 (57.7%)	144 (63.7%)	-
Mothers	127 (29.6%)	52 (26.0%)	74 (32.9%)	
Preferred sources of info on HIV/AIDS *				
Mothers	233 (53.6%)	103 (55.1%)	130 (60.5%)	-
Teachers	194 (44.6%)	80 (43.0%)	112 (52.1%)	

Notes: Mean age: 15.21 (1.08). * Proportions were not calculated; the two most cited sources were selected. N-values vary for different responses because of missing values.

### 3.2. Prevalence of Risky Sexual Behaviors

The prevalence of risky sexual behaviors among the participants is represented in Table 3. Overall, the proportion of learners who reported to have had sexual intercourse at the time of the survey was 113 (27.2%); significantly higher in boys than girls (boys = 33.8% *vs.* girls = 21.3%; χ^2^ (n = 416) = 8.29, *p* = 0.004). Among the sexually active learners, significantly more boys had sex before the age of 15 years (boys = 60.6% *vs.* girls = 31.9%; χ^2^ (n = 113) = 9.05, *p* = 0.003). The number of sexually active learners who reported more than one sex partner was higher in boys than in girls, though the association was marginally significant (boys = 50% *vs.* girls = 31.8%; χ^2^ (n = 102) = 3.39, *p* = 0.066). The association between gender and condom use for the last sexual intercourse and gender and the consistency of condom use was not statistically significant; however, the proportion of condom use was higher in boys (Table 3). The proportion of learners who reported to have undergone HIV testing was 13.0% in the total sample.

**Table 3:** Sexual behavior characteristics among the learners.

Characteristics	N	Total (%)	Boys (%)	Girls (%)	χ^2^	*p*-value
**Sexually active**						
Yes	113	27.2	33.8	21.3	8.29	0.004
No	303	72.8	66.2	78.7		
Early sex debut *						
Before 15 years	55	48.7	60.6	31.9	9.05	0.003
After 15 years	58	51.3	39.4	68.1		
Multiple partner *						
One	59	57.8	50	68.2	3.39	0.07
More than one	43	42.2	50	31.8		
Condom use for last sexual intercourse *						
**Yes**	57	53.3	50.8	41.3	0.95	0.33
**No**	50	46.7	49.2	58.7		
Regular condom use *						
Irregular	53	55.2	52.8	58.1	0.27	0.60
Always	43	44.8	47.2	41.9		
HIV testing *						
**Yes**	54	13	13.4	12.7	0.04	0.84
**No**	360	87	86.6	87.3		

Note: * Analysis excluded learners that were not sexually active.

### 3.3. Selected Predictors of Sexual Behaviors in Boys and Girls

Table 4 presents the relationship between selected predictors of sexual behaviors in boys and girls. Girls demonstrated a high level of self-efficacy belief (mean = 3.55, SD = 1.24) and negotiation skills (mean = 3.54, SD = 0.86) compared to boys in avoiding risky sexual behaviors. In contrast, boys had a higher risk of being influenced by friend’s sexual behavior (mean = 2.60, SD = 0.94) compared to girls. No significant gender differences were observed for HIV knowledge, communication skills and time perspectives. 

**Table 4:** Factors associated with sexual behaviors of boys and girls.

Factors	N	Boys M (SD)	Girls M (SD)	*t*-test scores	*p*-value
HIV knowledge	431	8.34 (3.03)	8.39 (3.11)	0.16	0.87
Self-efficacy	396	3.25 (1.22)	3.55 (1.24)	2.42	0.02
Communication	383	4.29 (0.94)	4.35 (0.79)	1.83	0.07
Peer influence	402	2.60 (0.94)	2.25 (0.90)	4.00	0.000
Negotiation	339	3.23 (0.77)	3.54 (0.86)	3.43	0.001
Fatalistic Present Time	350	2.75 (0.83)	2.69 (0.73)	0.70	0.49
Hedonic Present Time	323	2.77 (0.68)	2.82 (0.54)	0.83	0.41
Future Time	317	3.35 (0.74)	3.48 (0.64)	1.65	0.10

Note: N varies due to missing values.

### 3.4. Associations between Sexual Behavior Outcomes and Selected Predictors

As illustrated in Table 5, bivariate logistic regression was performed to assess the predictors that were associated with the likelihood of being sexually active, early sex debut and condom use for the last sexual intercourse. We did not present in Table 5 the association with the consistency of condom use and multiple partners, because no statistically significant associations were found with these variables. All of the selected covariates were fitted into the logistic regression model and the Hosmer–Lemeshow goodness of fit test (*p* > 0.05) indicated support for the models of being sexually active (χ^2^ = 5.77, *p* = 0.67), early sex (χ^2^ = 4.05, *p* = 0.85) and condom use (χ^2^ = 1.78, *p* = 0.64). If the association was significant, the analysis was then adjusted for gender.

As illustrated in Table 5, those who reported less resistance to peer influence were three-times more likely to report having had sexual intercourse (odds ratio = 3.01: 95 % CI (1.97–4.60)). When we adjusted for gender in the second model, boys were six-times more likely to report sex before the age of 15 (odds ratio = 6.60: 95 % CI (1.62–26.84)). Those with higher HIV knowledge were 1.22-times more likely to report using a condom (odds ratio = 1.22: 95 % CI (1.03–1.44)). Adolescents who reported easier communication with parents about sex were 63% less likely to report having had sex before the age of 15 (odds ratio = 0.27 (95 % CI: 0.08–0.91)). 

**Table 5:** Bivariate logistic regression of sexual behaviors.

Predictors	*Reporting Sexual Activity*			*Reporting Early Sex*			*Reporting Condom Use*		
	B	ORs	95% ORs	B	ORs	95% ORs	B	ORs	95% ORs
Gender (Boys)	−0.39	1.48	0.78–2.00	*1.89 ^a^*	*6.60 ^b^*	*1.62–26.84 ^b^*	−0.54	0.58	0.21–1.62
									
HIV knowledge	−0.04	1.04	0.93–1.15	−0.09	0.91	0.74–1.11	*0.20 ^a^*	*1.22 ^a^*	*1.03–1.44 ^a^*
									
Self-efficacy	0.12	1.13	0.85–1.50	0.22	1.25	0.72–2.18	−0.21	0.81	0.52–1.26
									
Peer influence	*1.10 ^a^*	*3.01 ^a^*	*1.97–4.60 ^a^*	0.64	0.53	0.23–1.20	0.38	1.47	0.76–2.82
									
Negotiation	−0.23	1.25	0.82–1.91	0.72	2.06	0.88–4.81	0.05	1.05	0.55–1.99
									
Communication 1	0.57	1.77	0.94–3.29	−*1.30 ^a^*	*0.27 ^a^*	*0.08–0.91 ^a^*	−0.25	0.77	0.25–2.41
									
Fatalistic	−0.30	0.97	0.49–1.89	0.28	1.32	0.38–4.49	−0.32	0.73	0.22–2.41
									
Future	0.12	1.23	0.57–2.22	−0.47	0.62	0.17–2.19	0.48	1.62	0.48–5.48
									
Communication 2	−0.21	1.24	0.82–1.87	0.31	1.36	0.66–2.81	0.27	0.81	0.52–1.26

Notes: ***^a^*** Values significant for crude odds ratio. ***^b^*** Values significant when adjusted for gender (girls were the reference group). Communication1: sexual communication with parents. Communication 2: communication with partners about sex.

## 4. Discussion

The increasing burden of adolescent risky sexual behaviors and the lack of definite factors that influence these risks are major challenges that hamper the successful implementation of HIV prevention programs for adolescents. This study describes the prevalence and factors that increase the risk and vulnerability to HIV infection among school-going adolescents aged 13–18 years in a rural town of South Africa.

The results of this study suggest that 27.2% of the learners have had penetrative sexual intercourse in their lifetime. This finding is consistent with a prior study among Grades 8–10 learners in South African schools, where 31.6% reported having had sex in their lifetime [21]. Although this rate is slightly higher than our study, the difference might be due to the fact that the study of Mojele and colleagues comprised both urban and rural schools. The possibilities of learners engaging in sexual behavior were much higher in boys, as other studies have equally observed [3,30,31]. Understanding the factors that increase adolescent risky sexual behaviors is critical for developing interventions that modify this risk and the resoluteness of health counselors and educators to identify and provide support for young people with heightened risk of HIV [32]. In our study, peer influence was a major predictive factor for learners who were sexually active. Learners who had less resistance to peer influence were more likely to be susceptible to peer pressure and three-times more likely to be sexually experienced compared to those who were less susceptible to peer pressure. This finding is consistent with a study that reported that young people who have sexually-experienced friends are more likely to be sexually active, too [11]. 

In addition, our study found that among learners who reported being sexually active, about 49% had experienced sexual intercourse before the age of 15 years, and this was significantly higher in boys (60.6%) than in girls (31.9%). The proportion of 49% who had engaged in early sexual intercourse in our sample is much higher than a study that found a lower rate of 27.3% [3]. This difference is likely due to the fact that adolescents older than 15 years were excluded from the above study. However, the trend observed in boys and girls in our study is consistent with other studies [3,33,34]. From the public health perspective, adolescents who engage in early sexual activities are less likely to use condoms and have more than one sex partner [35]. Our study found that gender difference and a lack of parental communication about sex are two major factors that predict early sexual debut in adolescents. Only 26.6% of the boys and 37.1% of the girls reported easy communication with parents on sex-related topics. These findings are consistent with studies that found that adolescents whose parents communicate with them about reproductive health and sex education are less likely to engage in risky sexual behaviors [36,37,38].

Regarding the condom use for the last sexual intercourse, only 53.3% of the sexually active learners reported having used condoms for their last sexual intercourse. However, the proportion of learners that used condoms in our sample is much lower than a study that reported a higher proportion of condom use in youth [4]. The explanation for this difference could be that learners in our study are in rural areas, where access to condoms and condom distribution are limited. Furthermore, the present study found HIV knowledge to be a strong predictor of condom use for the last sexual intercourse. This finding is consistent with studies that observed that HIV knowledge was associated with safer sexual practices, including consistent condom use [15,31]. An important aspect of our study was that girls had a higher self-efficacy belief and negotiation skills to avoid risky sexual behaviors. This is likely due to the fact that a significantly higher proportion of girls reported easier communication with parents on sex and never had sex compared to boys. One study observed that self-efficacy is easily enhanced in adolescents who have never had sexual intercourse compared to sexually active counterparts [39]. The implication of this finding is that interventions aimed at enhancing self-efficacy should be initiated in early adolescence before becoming sexually active.

## 5. Implications

Taken together, these findings add to the discussions around adolescent sexual behavior in several ways. Young people start engaging in sexual risk behaviors as early as 14 years or younger, and this period also offers a window of opportunity to commence age-appropriate sexuality education to either abstain from sex or practice safer sex. These findings should lend further support for comprehensive sexual health education, which is still lacking in some schools [40]. Although sexual abstinence in young people has been found to be the best approach to reduce the risks associated with risky sexual behavior, unfortunately, it is unlikely to make any meaningful impact, especially among youth who are already sexually active [41]. However, the promotion of condom use among young people has been met with stiff resistance in some countries, especially among faith-based institutions [42]. Implementing comprehensive sexuality education along with condom promotion among youth will therefore require supportive health-related policies from the government in schools. In South Africa presently, the age of consent at which young people can access HIV testing and contraceptives has been reduced to 12 years in order to eliminate any threat to sexual and reproductive health. It remains to be seen if schools have well-established youth-friendly health services for learners who would like to access these services. Thus, these findings are important for many reasons and assist in developing an effective intervention for young people in rural areas. Firstly, it is indicated that young people in rural areas have low knowledge of HIV; an effective school-based HIV prevention intervention must provide HIV knowledge information supported by skill-building activities to promote sexual abstinence and encourage condom use in learners. Secondly, school-based interventions must be developed in a way that the intervention activities include participants communicating with parents on sex-related matters during and after the intervention. Finally, a school-based intervention must develop peer-interaction among the learners to promote safe sexual behavior.

## 6. Limitation of the Study

The findings of the present study should be interpreted in light of the following limitations: (1) the study relied on cross-sectional data, thereby excluding the ability to make causal inferences; (2) data were collected through self-reported measures, and this is open to desirability bias, where learners could either over-estimate or under-estimate their true sexual behaviors. We randomized participants at the classroom level; therefore, we cannot assume the representativeness of the sample to the general adolescent population.

## 7. Conclusions

A greater number of school-going adolescents in rural areas are sexually active. Peer influence, especially for boys, is a factor that increases the preponderance of risky sexual behaviors in adolescents. Positively, adolescents with high knowledge of HIV infection are more likely to use condoms for every sexual encounter. There is a need to strengthen comprehensive sexual health education and youth-friendly HIV prevention strategies to promote abstinence or at least safe sex behaviors, especially among boys.
